# Supplementary material for: Hydraulic fracturing-induced delamination and extravasation extends medial damage beyond the false lumen in aortic dissection
Source: bioRxiv. 2026 May 16:2026.05.12.724712. Preprint. [Version 1] doi: 10.64898/2026.05.12.724712 (PMC13192997; doi:10.64898/2026.05.12.724712)
Supplement: Supplement 1 [file media-1.pdf]

SUPPLEMENTAL MATERIALS

Hydraulic fracturing-induced delamination and extravasation extends medial damage beyond the false lumen in aortic dissection

Alan Chou[1]*, Abdulrahman H.M. Hassab[1]*, Jay D. Humphrey[2,3], George Tellides[1,3,4†], Roland Assi[1,3,4†]

[1]Department of Surgery (Cardiac), Yale School of Medicine, New Haven, CT, USA.

[2]Department of Biomedical Engineering, Yale School of Engineering and Applied Science, New Haven, CT, USA.

[3]Program in Vascular Biology and Therapeutics, Yale School of Medicine, New Haven, CT, USA.

[4]Veterans Affairs Connecticut Healthcare System, West Haven, CT, USA.

*Denotes equal contribution as first author.

[†]Denotes equal contribution as senior author.

Supplemental materials: 2 tables, 7 figures

Correspondence: George Tellides, 10 Amistad Street, 337B, New Haven, CT 06520, USA. Phone: +1-203-737-2298, E-mail: george.tellides@yale.edu or Roland Assi, 330 Cedar Street, BB 225, New Haven, CT 06510, USA. Phone: +1-203-785-5000, E-mail: roland.assi@yale.edu

Supplemental Table 1: Characteristics of subjects with nondissected aortas*

	**Demographics**<br>Age, sex, race, ethnicity	**Ascending aorta**<br>Diameter, disease	**Cardiovascular**<br>Risk factors	**Aortic valve**<br>Function, leaflets
1.	28 yr, male, white, non-Hispanic	2.7 cm, no disease	Smoker, IVDU	No AI, tricuspid
2.	40 yr, male, white, Hispanic	3.6 cm, no disease	Smoker	No AI, tricuspid
3.	71 yr, female, white, non-Hispanic	2.8 cm, no disease	Smoker	No AI, tricuspid
4.	53 yr, male, white, non-Hispanic	3.0 cm, no disease	Smoker	No AI, tricuspid
5.	39 yr, male, black, non-Hispanic	2.8 cm, no disease	Smoker	No AI, tricuspid
6.	32 yr, male, white, Hispanic	3.4 cm, no disease	Smoker, cocaine use	No AI, tricuspid

*Ascending aorta specimens were procured with IRB approval from organ donors without known aortic disease ($n = 6$). Information was obtained from the electronic medical records and intra-operative observations. AI: aortic insufficiency, IVDU: intravenous drug use.

Supplemental Table 2: Characteristics of subjects with aortic dissection*

	**Demographics** Age, sex, race, and ethnicity	**Ascending aorta** Diameter and associated findings	**Aortic dissection** Extent and tear location	**Clinical presentation** Symptoms, onset to XCT, and operative findings	**Cardiovascular** Diseases and risk factors	**Aortic valve** Function and leaflet number	**Surgery** Aortic operation and associated procedures
1.	54 yr, male, white non-Hispanic	5.0 cm	Dissection from root to infrarenal aorta, tear in root	Chest pain, shock, 3 hr, tamponade	Alcohol abuse	Severe AI, tricuspid	Root/ascending/hemiarch replacement and antegrade TEVAR
2.	88 yr, female, white non-Hispanic	5.4 cm aberrant RSA	Dissection of ascending aorta and arch, chronic dissection distally, multiple tears of ascending aorta	Syncope, hypotension, 4 hr, tamponade	HTN, HLD, chronic type B aortic dissection	Mild AI, tricuspid	Root/ascending/hemiarch replacement
3.	63 yr, male, white non-Hispanic	4.2 cm	Dissection from ascending to iliac arteries, tear in proximal arch	Chest, back, and abdominal pain, 20 hr	HTN, obesity, family history of aortic dissection	No AI, tricuspid	Ascending/hemiarch replacement, aortic valve repair, and antegrade TEVAR
4.	72 yr, female, white non-Hispanic	4.8 cm	Dissection of ascending aorta, arch, and descending aorta, tear in lesser curve of ascending aorta	Chest pain, 7 hr, hemopericardium	HTN, HLD	Mild AI, tricuspid	Ascending/hemiarch replacement and aortic valve repair
5.	66 yr, male, white non-Hispanic	5.3 cm	Dissection of ascending and arch, tears in greater curve above STJ and proximal descending aorta	Chest pain, 7 hr, tamponade	HTN, obesity	No AI, tricuspid	Ascending/zone 2 arch replacement, antegrade TEVAR, and aortic valve repair
6.	78 yr, female, Pacific Islander, Hispanic	4.1 cm	Dissection from root to renal arteries, tear above right coronary artery	Chest pain, cardiac arrest, 5 hr, tamponade	HTN, HLD, DM, CAD, Afib, CKD, obesity	No AI , tricuspid	Root/ascending/hemiarch replacement and repair of coronary arteries
7.	81 yr, male, white non-Hispanic	4.8 cm	Dissection of root, ascending aorta, and proximal arch, tear in proximal arch	Chest and neck pain, 24 hr, tamponade	HTN, CKD, obesity	Mild AI, tricuspid	Ascending/zone 2 arch replacement
8.	50 yr, male, white non-Hispanic	4.6 cm	Dissection from root to abdominal aorta, tear in ascending aorta	Jaw, chest, and back pain, 5 hr	HTN, smoker	Mild AI, tricuspid	Ascending/hemiarch replacement and antegrade TEVAR
9.	77 yr, female, white non-Hispanic	4.2 cm	Dissection of root to mid-arch, tear in lesser curve of ascending aorta	Chest pain, 23 hr	HTN, HLD, MI, CAD, COPD	Moderate AI, tricuspid	Ascending/hemiarch replacement and aortic valve repair
10.	45 yr, male, white non-Hispanic	8.0 cm 6.4 cm root	Dissection of ascending aorta and arch, tear of proximal ascending aorta	Chest pain, shock, 5 hr, hemopericardium	None	Moderate AI, bicuspid	Root/ascending/hemiarch replacement and repair of right coronary artery
11.	59 yr, female, white non-Hispanic	4.0 cm	Dissection from root to descending thoracic, tear in mid-ascending aorta	Chest and back pain, 4 hr, tamponade	HTN, HLD, DM	No AI, tricuspid	Root/ascending/hemiarch replacement, bypass graft to right coronary artery, and repair of left main coronary artery

*Ascending aortic specimens were procured with IRB approval from patients with acute aortic dissection (*n* = 11). Information was obtained from electronic medical records, imaging studies, and intra-operative observations. Duration of aortic dissection is from the onset of symptoms to intra-operative cross-clamp time. XCT: cross-clamp time, AI: aortic insufficiency, TEVAR: thoracic endovascular aortic repair, RSA: right subclavian artery, STJ: sinotubular junction, HTN: hypertension, HLD: hyperlipidemia; DM: diabetes mellitus, CAD: coronary artery disease, MI: prior myocardial infarction, Afib: atrial fibrillation, CKD: chronic kidney disease, COPD: chronic obstructive pulmonary disease.

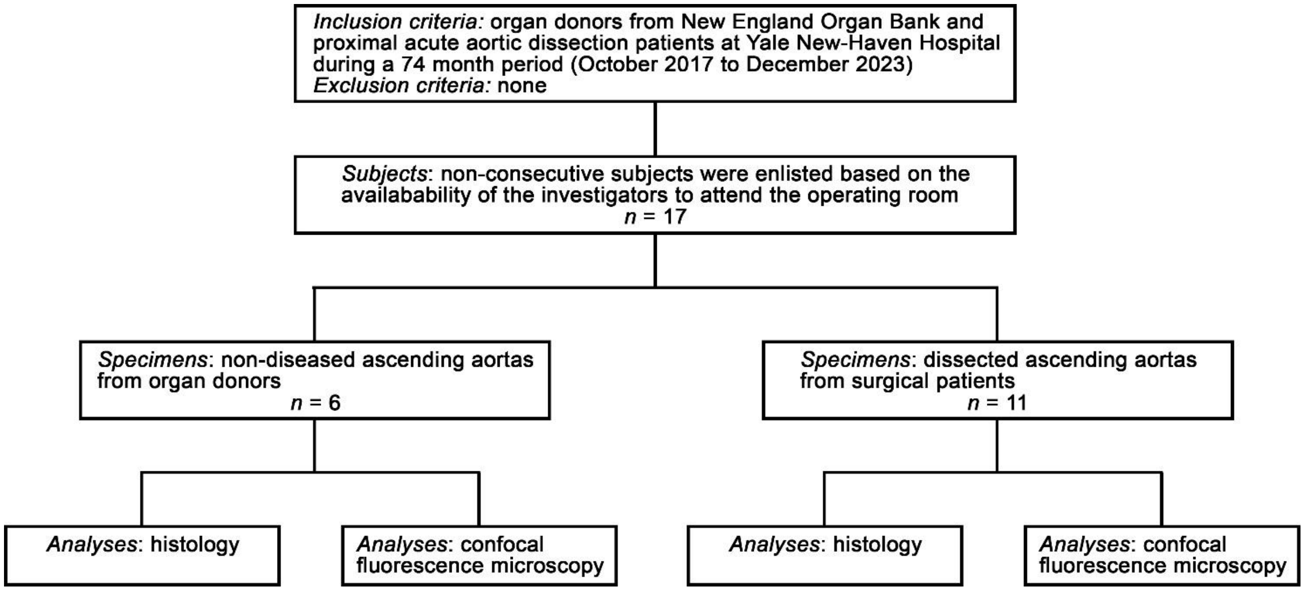

Supplemental Figure 1: Flow diagram of study design. Summary of inclusion/exclusion criteria, number of subjects enlisted, and number of specimens analyzed. Histology and confocal fluorescence microscopy were performed in 6 non-diseased aortas from organ donors and 11 acutely dissected aortas from surgical patients. Patients with aortic dissection compared to organ donors without aortic disease did not differ by sex (45.5% vs. 16.7% female, *P* = 0.33, Fisher's exact test), but were older (66.6 ± 13.8 vs. 43.8 ± 15.8 yr, *P* = 0.0153, unpaired t-test with Welch's correction) and had larger ascending aortas (4.9 ± 1.1 vs. 3.1 ± 0.4 cm, *P* = 0.0002, Mann-Whitney test); individual data shown in Supplemental Tables 1 and 2.

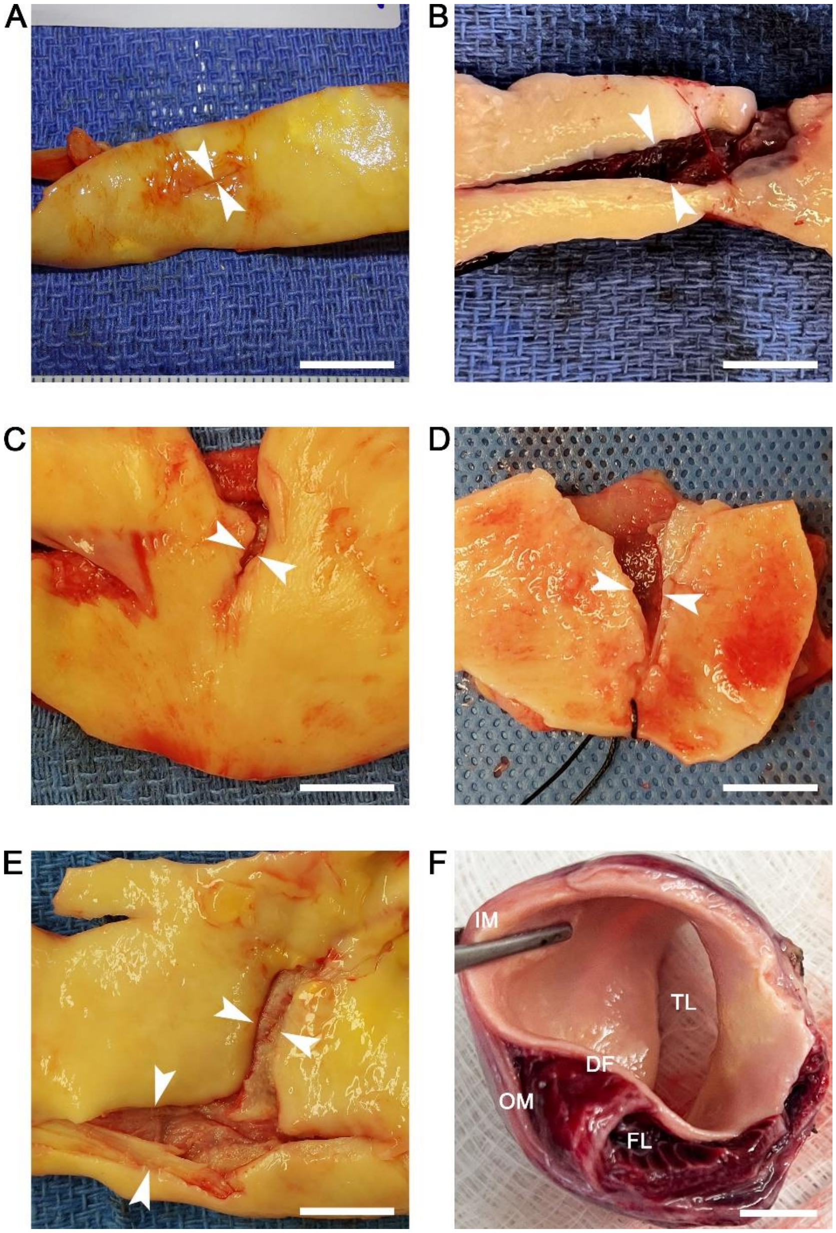

Supplemental Figure 2: Gross appearance of aortic dissection lesions. Specimens of proximal ascending aortas excised during surgical repair for dissection were examined in the operating room. (**A**) Transverse entry tear without retraction of edges. (**B**) Transverse entry tear with marked retraction of edges. (**C**) Longitudinal entry tear with mild retraction of edges. (**D**) Longitudinal entry tear with moderate retraction of edges. (**E**) Curvilinear entry tear with marked retraction of edges. (**F**) Transverse section through dissected ascending aorta showing true lumen (TL) and false lumen (FL) filled with thrombus; intact media (IM) that is not dissected bordering the true lumen, dissection flap (DF) consisting of intima and dissected inner media partitioning the true from false lumen, and dissected outer media (OM) with adventitia bordering the outer false lumen. Edges of entry tears are delineated by arrows. Orientation: proximal aspect of aorta at bottom and distal aspect at top (panels A–E), and intact media above and dissected media below (panel F). Scale bars = 1 cm.

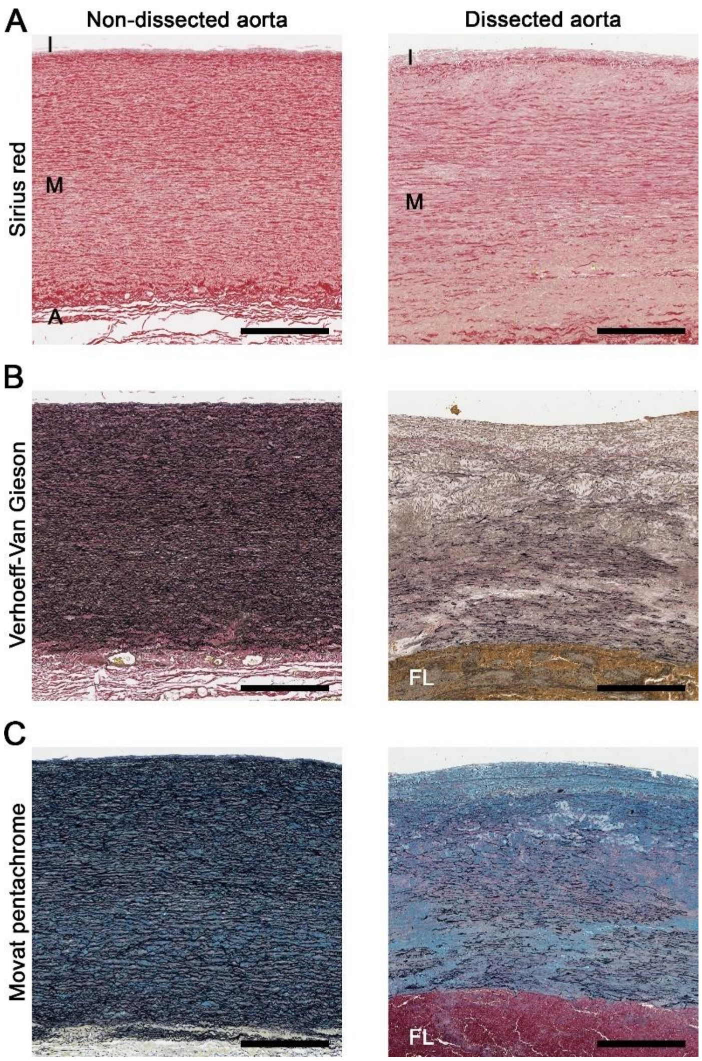

Supplemental Figure 3: Medial structure in non-dissected and dissected aortas. Histology of ascending aortas from an organ donor without medial degeneration (left panels) and acute dissection with pre-existing severe medial degeneration (right panels). (**A**) Sirius red stain labeling collagen (red) delineating the intima (I), media (M), and adventitia (A) layers. (**B**) Verhoeff-Van Gieson stain labeling elastin (black) and RBCs (yellow) delineating the false lumen (FL). (**C**) Movat pentachrome stain labeling elastin (black), SMCs and RBCs/thrombus (red), collagen (yellow), and glycosaminoglycans (blue). Orientation: luminal side top, adventitial side bottom. Scale bars = 500 µm.

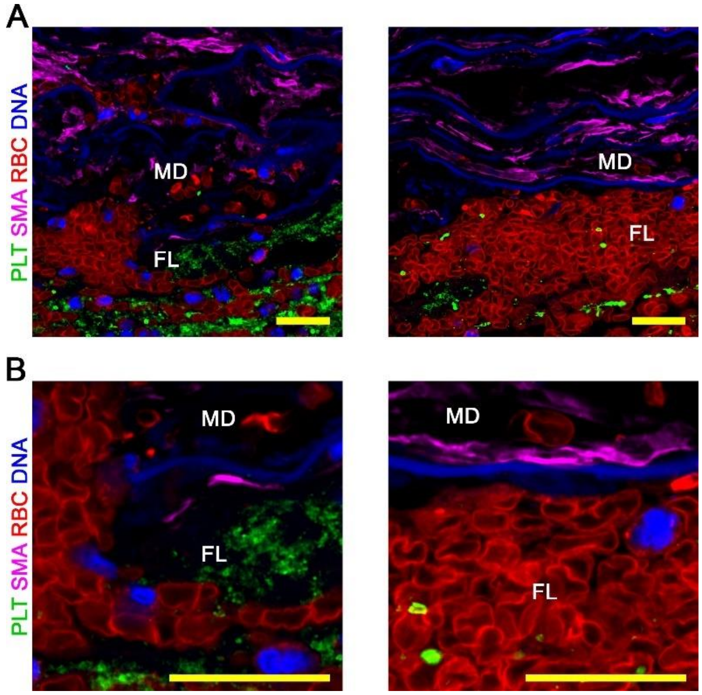

Supplemental Figure 4: Platelet distribution in aortic dissection lesions. Confocal fluorescence microscopy images of dissected aortas. (**A**) Lower and (**B**) higher magnification views for CD42b (green) identifying platelets (PLT), smooth muscle α-actin (SMA, magenta) identifying SMCs, CD235a (red) identifying RBCs, and DAPI (blue) labeling nuclei that show numerous platelets within the false lumen (FL) but few in adjacent delaminated laminae filled with RBCs, termed minor delaminations (MD). Elastic lamellae are faintly visible from blue autofluorescence. Orientation: luminal side top, adventitial side bottom. Scale bars = 20 μm.

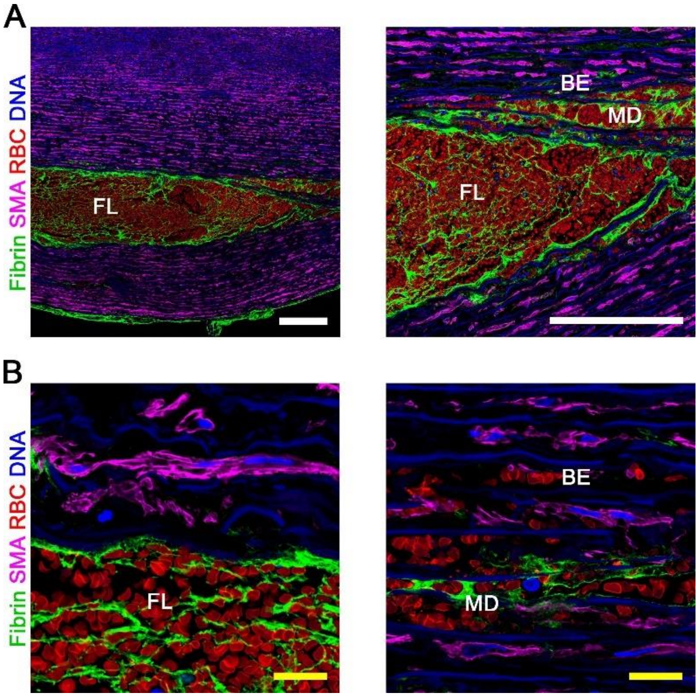

Supplemental Figure 5: Fibrin distribution in aortic dissection lesions. Confocal fluorescence microscopy images of dissected aortas. (**A**) Lower and (**B**) higher magnification views for fibrin (green) delineating thrombus, smooth muscle α-actin (SMA, magenta) identifying SMCs, CD235a (red) identifying RBCs, and DAPI (blue) labeling nuclei that show fibrin deposition within the false lumen (FL) and minor delaminations (MD) but rarely in non-delaminated laminae with RBCs, termed blood extravasation (BE). Elastic lamellae are faintly visible from blue autofluorescence. Orientation: luminal side top, adventitial side bottom. Scale bars: white = 250 μm, yellow = 20 μm.

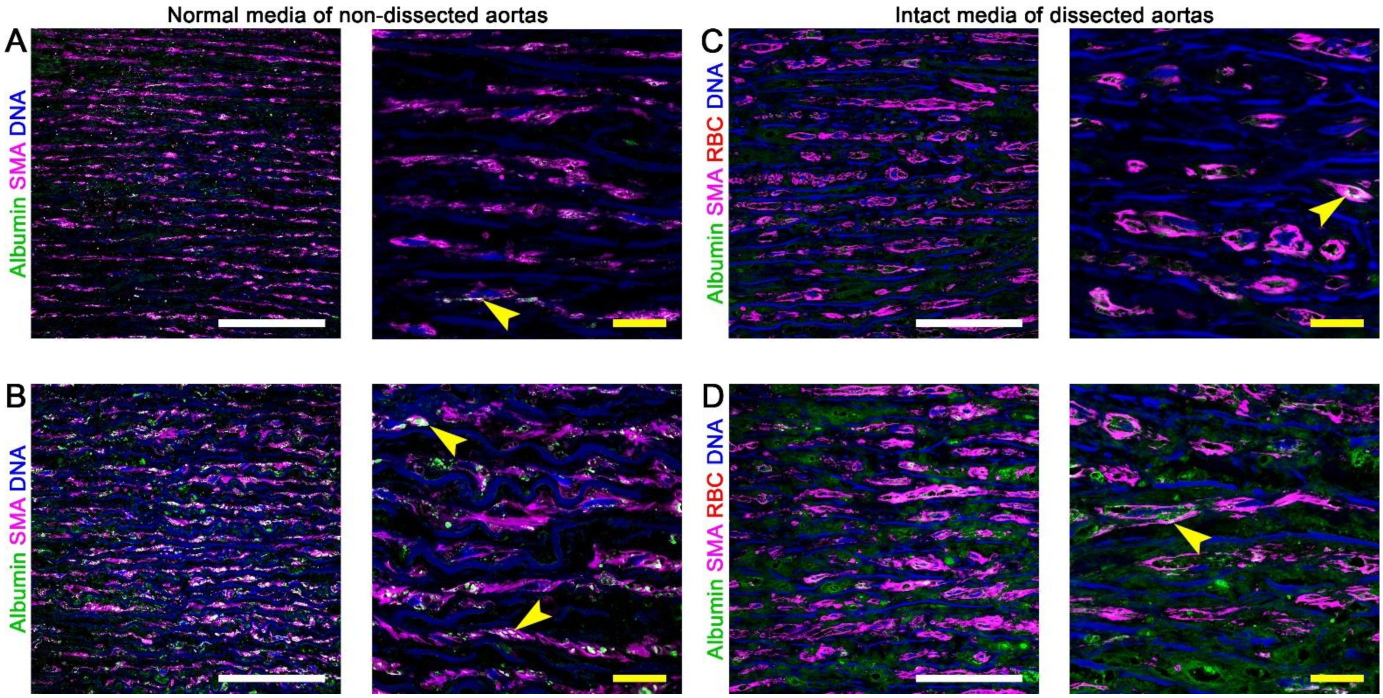

Supplemental Figure 6: Albumin distribution in non-dissected and dissected aortas. Confocal fluorescence microscopy images of aortic media. Lower (left panels) and higher (right panels) magnification views for albumin (green), smooth muscle α-actin (SMA, magenta) identifying SMCs, CD235a (red) identifying RBCs, and DAPI (blue) labeling nuclei show a spectrum of albumin accumulation from (**A**) minimal to (**B**) moderate in normal media of non-dissected aortas, and (**C**) minimal to (**D**) moderate in the intact media of dissected aortas. Overlay of intracellular albumin (green) and cytoplasmic smooth muscle α-actin (magenta) appears white (arrows). Elastic lamellae are faintly visible from blue autofluorescence. Orientation: luminal side top, adventitial side bottom. Scale bars: white = 100 μm, yellow = 20 μm.

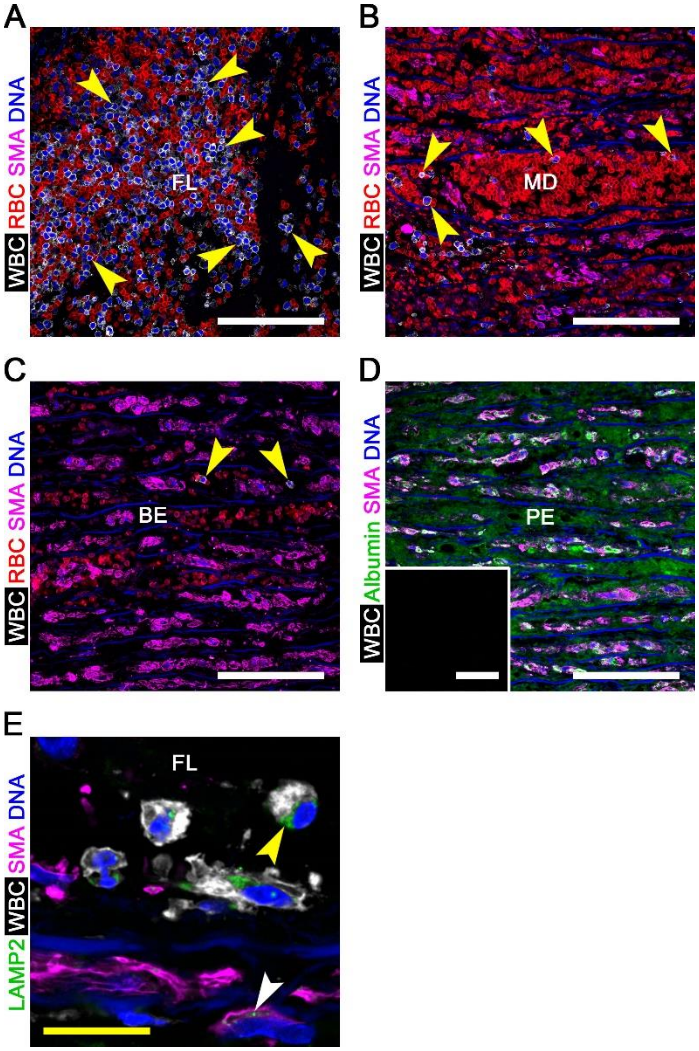

Supplemental Figure 7: Leukocyte distribution in aortic dissection lesions. Confocal fluorescence microscopy images of dissected aortas. CD45 (white) identifying leukocytes (WBC), CD235a (red) identifying RBCs, smooth muscle α-actin (SMA, magenta) identifying SMCs, and DAPI (blue) labeling nuclei showing (**A**) numerous leukocytes (arrows) in false lumen (FL), (**B**) frequent leukocytes (arrows) in minor delaminations (MD), and (**C**) infrequent leukocytes (arrows) in lesion areas with blood extravasation (BE). (**D**) Additional staining for albumin (green) showing absence of leukocytes in lesion areas with plasma extravasation (PE); fluorescence signal for CD45 was not detected (inset) though white color within SMCs results from overlay of magenta (SMA) and green (intracellular albumin) pseudocolors. (**E**) Alternatively, delineation of endolysosomes by lysosomal-associated membrane protein 2 (LAMP2, green), leukocytes by CD45 (white), SMCs by smooth muscle α-actin (magenta), and nuclei by DAPI (blue) verifies readily detectable organelles in mononuclear leukocytes (yellow arrow) but few in SMCs (white arrow). Elastic lamellae are faintly visible from blue autofluorescence. Orientation: luminal side top, adventitial side bottom. Scale bars: white = 100 μm, yellow = 20 μm.
